# Quality Assurance in Nursing Education: A Qualitative Study Involving Students and Newly Graduated Nurses

**DOI:** 10.3390/ijerph17010240

**Published:** 2019-12-29

**Authors:** Olga María López-Entrambasaguas, María José Calero-García, Ana María Díaz-Meco-Niño, José Manuel Martínez-Linares

**Affiliations:** Nursing Department, Faculty of Health Sciences, University of Jaén, Campus Las Lagunillas s/n, 23071 Jaén, Spain; mjcalero@ujaen.es (M.J.C.-G.); amdiaz@ujaen.es (A.M.D.-M.-N.); jmlinare@ujaen.es (J.M.M.-L.)

**Keywords:** education, nursing, nursing education research, nurses, qualitative research, students, nursing

## Abstract

*Background:* Assuring quality training for future nursing professionals is essential to preserving population health and socio-economic development. Quality assurance in the European Higher Education Area places students in a leading role to transform and improve higher education programs. Therefore, an innovative way of reviewing strengths and weaknesses of the nursing education program of a Spanish university has been developed. *Objectives:* The aim of this paper was to explore the perceptions and opinions of nursing students and newly-qualified nurses regarding the contents of the nursing curriculum in order to improve its quality. *Methods:* Descriptive and exploratory qualitative research was carried out involving 12 newly-qualified nurses and 12 student nurses. Semi-structured interviews and focus groups were performed. *Results:* Based on the thematic analysis, two themes emerged: improving clinical practices and reviewing the theoretical curriculum. *Conclusions:* Among the improvements suggested by the participants, the most relevant ones were establishing a clear structure of learning contents in the practicum, and redistributing the European Credit Transfer and Accumulation System ECTS credits in various courses of the study program. However, additional evidence is needed prior to proceeding with any changes.

## 1. Introduction

The regulatory framework of the European Higher Education Area (EHEA) brought about the implementation of quality assessment processes in higher education and other educational institutions, in order to provide societies with guarantees of educational effectiveness. Furthermore, Europe aims to enable mutual recognition among European educational systems and to become a world leader in higher education [1].

Higher education is considered fundamental in the socio-economic and cultural development of countries [1]. Likewise, population health is the base of socio-economic progress of countries and properly trained nursing professionals are in charge of taking care of the people’s health. Consequently, quality training of nurses is crucial to eventually achieving such goals.

In 2005, the Standards and Guidelines for Quality Assurance in the European Higher Education Area (ESG) were published [1]. A subsequent revision of the ESG was launched and adopted in 2015 [2], where teaching and learning quality assurance was established as the main goal.

One of the four principles of quality assurance in the EHEA includes “to take into account the needs and expectations of students” [2] (p. 8). Likewise it is stated that the design of study programs should involve students (and other stakeholders) and consider experts’ opinions. Additionally worth-highlighting is the need established upon this document to regularly review and revise the study programs [2] (p. 15).

Nursing education and training has undergone major changes in Europe since the implementation of the Tuning Educational Structures in Europe Project [3]. In Spain, the adaptation of the nursing curriculum to the new standards meant both a quantitative change and qualitative improvement towards professional development. Higher nursing studies used to consist of a 3-year degree between 1977 and 2008 [4]. This academic curriculum was modified according to the regulations derived from the Bologna Process, so that the new design would enable one to acquire the competences required for nursing work.

In our context, higher education institutions must guarantee continuous improvement of their teaching [5], through improvement policies and Internal Quality Assurance Systems (IQAS). The design of the IQAS of the Faculty of Health Sciences of the university under study (University of Jaén) was certified in 2009 [6]. Universities eligible for IQAS certificates must “provide training that guarantees the needs and expectations of students and society” and prove that “the main stakeholders are involved, primarily the students” [7] (p. 5).

Consequently and according to the standards and guidelines of the internal quality assurance of the EHEA and Spain, the Nursing Department of our university decided to implement a new procedure to review and revise the study program, involving undergraduate and graduate nurses. This research comprised of four objectives: (1) to explore and comprehend the perception of newly qualified nurses (NQN) about their competency training and acquisition after they have finished studying at university [8]; (2) to understand the perception of both nursing professionals and fourth-year students of nursing on the connection between the knowledge acquired throughout the degree and the professional healthcare practice [9]; (3) to understand their perception of the lecturers’ and clinical preceptors’ effectiveness [10]; and the fourth and the last one, which is presented in this paper, and it is to explore students’ and newly-qualified nurses’ (NQN) perceptions and opinions concerning the nursing study program, in order to guarantee their expectations, needs and, if necessary, to improve the theoretical/practical program.

## 2. Materials and Methods

### 2.1. Design

An exploratory and descriptive qualitative study was developed in a public university of Spain. We adopted this methodology, as we considered it the most appropriate one in order to investigate an unexplored area [11]. Qualitative methods are appropriate when issues are complex and include concepts that are difficult to measure [12].

### 2.2. Participants

The candidates who were to participate in the study were NQN who had completed the bachelor’s degree and 4th-year nursing students of the university under study. A convenience sampling was carried out using the following inclusion criteria:

(1) For NQN, having 3–12 months of work experience in Spain and having completed their university education in the years 2017, 2016, or 2015. These criteria were established by considering the possibility to find theory-practice gap results in the most recent alumni.
(2) For 4th-year nursing students, completion of all the theoretical training of the bachelor’s degree and six out of seven internships.

The NQN were contacted by email and were provided with information on the aim of the study, anonymity, confidentiality and data collection. Students were likewise invited to participate via email and were also provided with the same information once the results from the research with the NQN were obtained. A total number of 16 NQN showed an interest in participating. A total number of 12 NQN and 12 students were selected to join the sample.

### 2.3. Data Collection

Data were collected between March and April 2018 through 12 semi-structured individual interviews of the NQN. Nine of them were held face-to-face in a university meeting room. For three participants who were geographically dispersed, the interviews were conducted via Skype. Data collection stopped when data saturation was achieved [13] (p. 587). Saturation of data was reached with 12 out of 16 NQN who were interesting in participating. The total number of students who were interviewed in groups was the same number of students who were primarily interested in being part of the study. It was not needed to increase the number of these participants as additional new information was not attained.

A deep review of the international literature was done prior to the drafting of the interview guide [14]. It was eventually configured to around three pre-specific categories: theoretical training, clinical training, and reality of healthcare. For achieving the purpose of this paper, questions were made only regarding clinical and theoretical training (Table 1). The interviews were audio recorded and later transcribed verbatim, where each one lasted between 45 min and 1.5 h. The interviews were conducted by two female researchers and one male researcher using direct dialogue [15] and the building of rapport to confirm validity [16]. In order to control bias, the interviewers were researchers/lecturers who had not taught the NQN when they were students at the university and, therefore, interviewees and interviewers did not know each other.

The 4th-year students were divided into two focus groups (FGs) of six participants in September 2018. The FGs were carried out by two students who were collaborating with and learning in the research group to which the researchers belonged. The researchers did not perform the group interviews themselves in order to minimize bias, as they had been teaching the participants the previous year. The FG interviews were recorded and lasted between 1 and 1.5 h. The students who performed the group interviews also transcribed them, protecting the anonymity of the participants before delivering them to the researchers for analysis.

### 2.4. Data Analysis

A qualitative thematic analysis was performed using the Atlas. ti software version 7 (Jaén, Spain) for Windows (Microsoft^©^) ATLAS.ti. 7 software. Two of the researchers independently analyzed the transcriptions, following the step-by-step guide presented by Braun and Clarke [17]. Interviews were read and re-read “in an active way” [17] (p. 16) to produce an initial list of ideas. Afterwards, systematic coding was applied to meaningful data clusters and, subsequently, the codes were sorted into potential subthemes and themes. In the next step, the initial themes and subthemes were reviewed and refined. To ensure reliability, the instructions suggested and presented by Nowell et al. [18] were followed. Peer debriefing and triangulation were followed to generate the final codes and throughout the whole process of connecting subthemes and reviewing-defining-naming themes. Detailed notes, diagramming of themes and subtheme connections and documentation of the team meetings were assured [18].

### 2.5. Ethical Considerations

The Human Research Ethics Committee of the university approved this investigation (Reference: JUL.18/1.PRY). Free and informed written consent was obtained before the data were collected. Confidentiality and anonymity were assured throughout. In addition, participants were informed that they could withdraw from the study if they wished to do so.

## 3. Results

**Figure 1:**
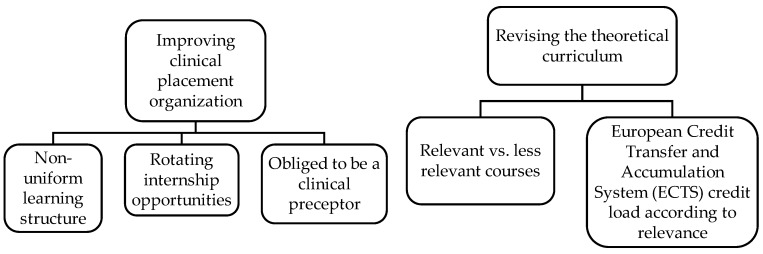
Themes and subthemes. Source: prepared by the authors.

The findings were framed within two themes and six subthemes that reflected the experiences and perceptions of students and NQN (Figure 1).

### 3.1. Improving Clinical Placement Organization

The analysis of the interviews showed participants’ dissatisfaction with some aspects of the practicum: learning, length, clinical preceptors, and evaluation.

#### 3.1.1. Non-Uniform Learning Structure

During the bachelor’s degree Practicum 1, students must practice the most basic nursing care, such as patient hygiene, together with the assistant nursing staff. The interviewees said that some students fulfilled this assignment, and others did not. The latter would directly perform rather technical activities that they would carry out when working as qualified nurses. Additionally, not spending a lot of time with nursing assistants was valued positively:

“I clearly knew my competences in Practicum 1 and that I was going to be with the nursing assistants, and I think it’s OK, […] but, when I started at the hospital, I had a nurse preceptor who used to laugh: ‘do you really want to be with the assistants? Come with me! Draw blood!’. I would say: ‘I cannot!’, and she replied: ‘Don’t talk nonsense! Nobody is looking’—‘They let me do everything, I was very lucky” (I3).

“It depends on the preceptor. I was in […] and my preceptor let me draw blood and give injections from the very first day, although I understand that we were doing Practicum 1… Anyway, I know that other preceptors did not let students approach patients and made them stay in the background…” (S7).

The following interviewee describes two completely different learning experiences during her practicums. During the first practicum, she related the feeling of not knowing exactly what competences she would acquire, and that those would depend on the assigned clinical preceptor. On the other hand, her second experience was about her placement in another clinical service, where her learning was quite structured and she became a part of that process:

“Before starting the operating room practicum, we did not know what specific competences we were going to learn. We just had an idea of what is done in that unit. For instance, we knew we were going to prepare the instrumentation, to later find out that we also needed in-depth knowledge about anesthetics, which was not addressed by any document… So, the competences often acquired will depend on your preceptor and, especially, on the preceptor’s view of the service which they work at” (I8).

“On the first day, we were given a sheet which included what we had to check daily together with our preceptor. Then, every day, I would perform as my preceptor generally would, but with special emphasis on the tasks to be performed each time; thus, at the end of the shift, nothing was overlooked. Also, each day before the start, we had to read some literature related to the subject. Maybe this was a bit excessive, but you could catch a glimpse of what you would learn by having some guide notes at hand on the things to be checked and it also encouraged me to look for more info on the subject” (I8).

#### 3.1.2. Rotating Internship Opportunities

Most of the participants agreed on an insufficient number of clinical placement hours. The participants thought that there should be more rotating internships per service and more services available:

“What I would increase indeed is the number of placements, because the range would be broadened, and we could attend a greater number of specialized services” (S8).

The following interviewee regretted not having had the chance to do any practicum related to maternal and child health:

“I would have liked to do a greater number of placements, because I did not work in some services, such as maternity or pediatrics” (I5).

A suggestion made by many of the interviewees was that the rotating internships should be longer:

“You only spend 25 days per service, so when you are just getting adapted to the patients, the workflow, the organization… you have to leave the placement” (I2).

However, a different perspective was expressed by one of the nurses interviewed:

“Do you think you had enough clinical practice hours?—I think so, I think it is fine to learn the most important things” (I11).

#### 3.1.3. Obliged to be a Clinical Preceptor

Questions were asked on satisfaction with the learning experience and positive and negative opinions about the preceptors. Most of the participants mentioned at least one bad experience or something negative related to any of the seven preceptors they had, although not all of them described what happened. The negative experiences reported had the same origin/background: the clinical preceptor was forced to perform as such:

“During one practicum, I did have problems with a preceptor. When I arrived and introduced myself, she said: ‘You, student? I said I did not want a student’.—[…] She did not let me do almost anything during the whole rotating internship” (I3).

“What I see is that they feel obliged to do it, many of them do not want to be preceptors” (S5).

“Lack of enthusiasm, little empathy, or little warmth…” (I4).

An interesting issue that emerged in the analysis of the interviews was that most of the interviewees rated their relationship with their clinical preceptors as very positive or positive. However, when asked if they thought that their preceptors were well-prepared to perform such a role, their assessments were worse:

“In general, how would you assess your relationship with your preceptors?—I think it was very good, because most of them considered us colleagues, and that made us feel good […].—Do you think that the preceptors assigned to you were well-prepared to perform such a role?—Hmm… no, not always. I would say fifty percent according to my experience” (I6).

“How would you assess your relationship with your preceptors?—Good in general, I have never had any problem of confrontation or felt uneasy […]. Do you think they were well-prepared to perform such a role?—Hmm… not hundred percent, but sixty or seventy percent, yes… They might be a little overwhelmed by the role” (I12).

### 3.2. Revising the Theoretical Curriculum

Proposals for revision of the teaching load in some courses and some constructive criticism emerged from the analysis of the interviews, in order to develop curricular content with a rather practical component.

#### 3.2.1. Relevant vs. Less Relevant Courses

There was a fairly broad consensus on what a “relevant course” was. The interviewees valued more positively the theoretical contents related to techniques and those required to face their day-to-day work:

“At the end of the day, we, low-level nurses, do not do management work, and the people chosen for management are required to specialize. Therefore, that course could definitely be removed” (I12).

“‘Alternative Care’ and ‘Culture, Gender, and Health’ courses should be removed because they do not contribute much” (I9).

“I think there are some shortcomings in ‘Clinical Nursing’ and ‘Physiology’; more contents should be taught” (I11).

#### 3.2.2. European Credit Transfer and Accumulation System ECTS Credit Load According to Relevance

Following the comments described in the previous sub-section, most participants negatively judged the fact that all courses were the same number of ECTS credits. They thought that the “most relevant courses” must be assigned more credits than those classified as “less relevant”:

“I find it unbalanced that ‘Human and Therapeutic Relationships’ and ‘Culture and Gender’ courses are six credits, as well as ‘Clinical Nursing’, for instance. Clinical Nursing, in my opinion, falls rather short and it would be more interesting if it were longer and other courses were shorter. I think we have gone from a 3-year degree to a 4-year degree and nothing has been done to give more relevance to the courses that really matter” (S1).

There were also opinions that directly addressed the elimination of courses, as shown below:

“‘Child and Teenage Nursing’ is also important, as well as ‘Emergency’, but I think I would take ‘Complementary Care’ away, or I would make it as an elective course” (I2).

Another option proposed by some participants was to merge similar courses that, as they explained, repeated content:

“‘Human and Therapeutic Relationships’ could be merged with ‘Psychosocial Sciences’” (I1).

“The best would be to merge some courses in order to shorten the content load” (S8).

## 4. Discussion

Participants showed overall satisfaction with the teaching received at university. However, they highlighted some major and necessary issues to improve the quality of the nursing academic curriculum.

A vast majority of the interviewees reflected that the work carried out by the preceptors was welcomed. However, the way they experienced the learning and performed the tasks depended on the preceptor assigned to them, and it could seem that it was not based on structured educational content. The result of the final analysis made on their statements was that the learning process in placements depends on “being lucky with the nurse preceptor assigned”. There could be then a certain lack of coordination between the university and the clinical placements, as well as a disparity in the organization of the teaching-learning process, resulting in the students’ dissatisfaction. Fernández-Sola et al. [19] pointed out the necessity of fostering coordination between the university and the participating healthcare centers in order to improve the quality of the clinical practice training model. On the other hand, according to another Spanish study by Fuentes-Pumarola et al. [20], the students also raised the subject of having good or bad luck with nursing instructors to guide their learning experience. Shivers et al. [21] stated the importance of a high-quality practice learning experience to foster learning outcomes.

Meeting students’ expectations and the length of the clinical placement experience contributes to authentic learning, according to the integrative review of Walker et al. [22]. Practicums are the most valued learning scenarios among the interviewees, over theoretical and theoretical-practical lessons. In healthcare settings, students have the opportunity to develop professional skills and get closer to the professional world. Actually, the strongest critics of those who were interviewed were those already in the labor market. They suggested that more hours should be incorporated into clinical placements so that they (nurses) could feel better prepared when facing the reality of being nurses. This higher dissatisfaction in graduate nurses in contrast with students was also found by Milton-Wildey et al. [23] and Günay and Kilinç [14]. Work readiness is an area of concern still under research in our field and our findings echoed those of Meyer et al. [24] and Milton-Wildey et al. [23] in that nurses felt unprepared to enter working life. In a recent review [25], it was clearly highlighted that the educational factor is one of the most important contributors to work readiness. This review also reported that a sufficiently long clinical practice period was meaningful and essential for the achievement of competences. However, there was a lack of consensus around what the appropriate length of a clinical nursing training program should be. According to the Spanish regulation [26] the amount of ECTS credits of the Practicums and the Degree’s Final Project must add to 90. In our university, 84 ECTS credits are assigned to the Practicum (2100 h), where clinical placements are 56.4 ECTS credits (1410 h). The rest of ECTS credits, up to 84, correspond to other activities (e.g., Creation of a portfolio). There is no consensus even within the Spanish territory about the number of actual hours of placement in clinical settings. The most recent legislation incorporated into the Spanish legal system [27] includes the latest European guidelines on nursing education [28], which also fails to specify the exact teaching load of the clinical training program. However, Article 42 stipulates that clinical training must be at least 2300 h.

The nurse preceptor’s competence plays a crucial role in the students’ learning process [29,30]. Our participants indicated that some of the instructors did not perform their function well. The reasons were related to several aspects: the teaching methodology, the compliance with respect to the nurse’s role learned in the university, and the possibility of practicing techniques and procedures. The interviewees were disappointed in seeing nursing staff who did not provide care based on the latest scientific evidence, as they learned in university. Several authors found differences between what students learned at university and what they later saw in the healthcare settings [9,14,20,31,32]. The literature also suggests that idealized care and actual care act like two opposing forces that graduates try to counterbalance [33,34].

Some participants made quite critical comments referring to the theoretical courses. For them, “not all courses have equal relevance”, considering some courses more relevant and complaining about all courses having the same credit load. They undoubtedly deemed more relevant and useful those they could see as clearly connected with the day-to-day nursing work and those that required practical skills and techniques; these results match those of Fuentes-Pumarola et al. [20]. Kermansaravi et al. [32] found that students liked theoretical contents that had applied value; otherwise studying them was a waste of time. We can state that there was a preference for biomedical courses, which could contrast/contradict the long struggle of nursing to carry out the conceptual and theoretical framework change in nursing training, as opposed to the traditional biomedical model [35]. In any case, the credit load is revisable and susceptible to modification, since the number of credits that each course must be is not stipulated by any Spanish law [5,26,27]. Nowhere does the European Directive 2013/55 [28] contain specific mention to this issue, it is only stated that “[…] the theoretical training represents at least one third […] of the minimum duration of the training”. No regulation establishes which courses should have more credit load according to their relevance. We have not found in the literature any investigation that approaches this matter of amount of credit *hours for relevant courses* in nursing education. A recent Swiss study [36] explains the step-by-step revision process of a Master’s degree in Nursing Sciences curriculum in detail, however this specific concern is not approached. On the other hand, Van de Mortel and Bird [37] mention that duplications found in the curriculum review were removed, although our matter was not addressed by them.

In the same vein, Pajnkihar et al. [38] (p. 243) reported that in healthcare systems more emphasis was placed on diagnostics and therapeutics than on the psychological, spiritual, and social aspects of care. This is particularly challenging for nursing education, and raises a few questions of concern that require deeper reflection and further research in our context: (1) Is the change from the biomedical model to the holistic ineffective? (2) Should we, lecturers, keep on teaching content which is not actually put into practice? How could it be balanced? (3) The importance of the traditional biomedical model has declined at the academic level but has not at the clinical level, why? Should the organization/structure of the health system be modified?

### Limitations

This study offers a detailed portrait of students and NQN’ opinions, and provides information that may be relevant to making changes in the study program. However, it could be possible that students and NQN who had a more negative experience at this university may have been more motivated to participate in the study. Therefore, further research would be recommended that include clinical preceptors, stakeholders and experts in order to contrast opinions with these results.

## 5. Conclusions

This university demonstrated adherence to best practice in seeking the views of students and nurses, as part of the process of improving the nursing curriculum. After the analysis of the research results, highly important items susceptible to modification were detected, such as the insufficient hours of clinical training. Regarding the clinical training program, students seem to be negatively affected due to the lack of a clear structure in the learning content and the fact that many nurses are forced to be preceptors. With respect to the theoretical content of the curriculum, the participants reflected on what they thought could be improved and what needed to be changed to foster usefulness of such content in the workplace and feeling competent when successfully performing their functions.

Currently, the research team is setting the research process with other stakeholders in motion, as recommended within the limitations part of this paper.

## Figures and Tables

**Table 1:** Main questions of the interview guide.

Pre-Established Categories	Main Questions
Clinical training	Are you satisfied with your learning experience in the Practicum? What is your opinion about the organization of the Practicum? Did you find any differences in the organization of the learning among the diverse clinical placements?
Theoretical/Academic training	Are you satisfied with your learning experience in the classes? Which courses do you think are most important? Can you explain why? Which courses do you think are less important? Can you explain why? Do you think the credit load of the courses is adequate? Would you suggest any improvement?
